# Genetic Analysis and Literature Review of *SNCA* Variants in Parkinson's Disease

**DOI:** 10.3389/fnagi.2021.648151

**Published:** 2021-08-12

**Authors:** Yi Guo, Yan Sun, Zhi Song, Wen Zheng, Wei Xiong, Yan Yang, Lamei Yuan, Hao Deng

**Affiliations:** ^1^Department of Medical Information, School of Life Sciences, Central South University, Changsha, China; ^2^Center for Experimental Medicine, The Third Xiangya Hospital, Central South University, Changsha, China; ^3^Department of Neurology, The Third Xiangya Hospital, Central South University, Changsha, China; ^4^Cancer Research Institute, Xiangya School of Medicine, Central South University, Changsha, China; ^5^Disease Genome Research Center, Central South University, Changsha, China

**Keywords:** Parkinson's disease, *SNCA* gene, genetic analysis, whole-exome sequencing, Sanger sequencing, variant

## Abstract

Parkinson's disease (PD) is the fastest-growing neurodegenerative disorder. Aging, environmental factors, and genetics are considered as risk factors. The alpha-synuclein gene (*SNCA*), the first pathogenic gene identified in a familial form of PD, was indisputably involved as a heritable component for familial and sporadic PD. In this study, whole-exome sequencing and Sanger sequencing were performed to evaluate the association between the *SNCA* gene variants and PD. The genetic data of 438 clinically diagnosed patients with PD and 543 matched control populations of the Han Chinese were analyzed. The literature review of *SNCA* variants for 231 cases reported in 89 articles was extracted from the PubMed and the Movement Disorder Society Genetic mutation database. No potentially causative variant(s) in the *SNCA* gene, excepting two single-nucleotide nonsynonymous variants c.158C>T (p.A53V, rs542171324) and c.349C>T (p.P117S, rs145138372), were detected. There was no statistically significant difference in the genotypic or allelic frequencies for either variant between the PD group and the control group (all *P* > 0.05). No copy number variants of the *SNCA* gene were detected. The results of this study suggest that the variants in the exons of the *SNCA* gene may have less or no role in the development of PD in the Han Chinese populations. The literature review suggests that psychiatric signs and cognitive decline/dementia were more common among patients with *SNCA* duplication or triplication (psychiatric signs: χ^2^ = 7.892, *P* = 0.005; cognitive decline/dementia: χ^2^ = 8.991, *P* = 0.003).

## Introduction

Parkinson's disease (PD), first reported by Dr. James Parkinson in his *An Essay on the Shaking Palsy* in 1817 and named by Jean-Martin Charcot, is the fastest-growing neurodegenerative disorder with an age-related prevalence of ~3% among the population aged above 75 years and 4–5% of people older than 85 years (Hernandez et al., 2016; Emamzadeh, 2017; Billingsley et al., 2018; Hopfner et al., 2020). It is conservatively estimated that the PD cases will be 2-fold, i.e., from 6.2 million in 2015 to 12.9 million by 2040 (Dorsey and Bloem, 2018). Neuropathologically, PD is characterized by dopaminergic neuron loss in the substantia nigra pars compacta and insoluble alpha-synuclein-containing Lewy bodies formation in the remaining nigral neurons (Cook Shukla et al., 2004; Billingsley et al., 2018). Although the precise etiology has not been completely elucidated, advanced age, environmental influences, and genetics are thought to be the risk factors (Langston et al., 2015; Dorsey and Bloem, 2018). PD motor deficits include any or all of the following: bradykinesia/hypokinesia/akinesia, resting tremor, muscular rigidity, postural abnormality, gait disturbance, and freezing. The clinical pictures also include a range of nonmotor manifestations such as olfactory dysfunction, autonomic impairment, cognitive decline, and neuropsychiatric disturbance (Postuma et al., 2015; Liu and Le, 2020).

Although ~5–10% of PD causation has been attributed to different monogenic causes, sporadic PD can be related to various additional genes and susceptibility loci (Balestrino and Schapira, 2020; Toffoli et al., 2020). Several technologies, including the high-throughput techniques such as whole-exome sequencing (WES) and whole-genome sequencing, as well as machine learning and single-cell RNA sequencing, have discovered a great number of genetic risk factors in PD (Deng et al., 2018; Blauwendraat et al., 2020). To date, at least 23 genes related to parkinsonism and a number of independent risk signals have been identified (Langston et al., 2015; Deng et al., 2018; Nalls et al., 2019; Blauwendraat et al., 2020).

The alpha-synuclein gene (*SNCA*) was indisputably considered as the first pathogenic gene responsible for autosomal dominant PD, supported by the fact that its protein aggregation is thought to be the primary pathological hallmark of the patients, though only a few mutations were identified (Polymeropoulos et al., 1997; Fields et al., 2019). In this study, WES and Sanger sequencing were performed to evaluate the association between *SNCA* gene variants and PD, and the literature review of *SNCA* variants was conducted.

## Materials and Methods

### Study Subjects and Clinical Evaluation

The WES data of the *SNCA* gene were extracted from the internal PD-control database. A total of 981 Han Chinese participants from mainland China were recruited, including 438 clinically diagnosed patients with PD (male/female: 49.54%/50.46%, mean age: 59.19 ± 11.46 years, and mean age of onset: 55.67 ± 12.35 years) and 543 age- and sex-matched controls (male/female: 50.09%/49.91%, mean age: 58.11 ± 13.61 years) who had neither PD clinical symptoms nor a family history of PD. Among them, 438 patients with PD were consecutively recruited into this study, of which 308 were unrelated sporadic cases and 130 had a family history of PD. All PD individuals were evaluated by two experienced neurologists from the Third Xiangya Hospital, Central South University (Changsha, China) and were diagnosed with PD based on the official International Parkinson and Movement Disorder Society Clinical Diagnostic Criteria for PD (Postuma et al., 2015). The protocol was approved by the Institutional Review Board of the Third Xiangya Hospital, Central South University and was conducted in accordance with the tenets of the Declaration of Helsinki. Written informed consents were obtained from all participants. Moreover, a comprehensive literature review was performed to assess the associations between phenotypes and *SNCA* gene variants by PubMed (https://pubmed.ncbi.nlm.nih.gov/) and the Movement Disorder Society Genetic mutation database (MDSGene, https://www.mdsgene.org/). The following key search terms were used: “*SNCA*,” “alpha-synuclein,” “PD,” “Parkinson's disease,” “Parkinson disease,” “point mutation,” “mutation,” “variant,” “multiplication,” “duplication,” and “triplication.”

### WES and Sanger Sequencing

Genomic DNA (gDNA) was harvested from peripheral blood leukocytes according to the standard phenol–chloroform extraction as previously described (Yuan et al., 2015). WES was conducted on the gDNA to construct an internal PD-control database as previously described (Xiang et al., 2019). Frequencies of variants, such as single-nucleotide polymorphisms (SNPs) and insertions–deletions, were assessed using several public databases, such as the 1000 Genomes Project, the Single Nucleotide Polymorphism database (version 154), the Exome Aggregation Consortium, the Genome Aggregation Database, and the China Metabolic Analytics Project database (Xiang et al., 2019; Cao et al., 2020). The variant with an allele frequency over 1% was further filtered out, and the bioinformatics prediction software, such as Sorting Intolerant from Tolerant (SIFT), Protein Variation Effect Analyzer (PROVEAN), Polymorphism Phenotyping version 2 (PolyPhen-2), and MutationTaster, were utilized to evaluate the potential effects on the function and structure of the alpha-synuclein protein. The potential pathogenicity of identified variants was further assessed using the pathogenicity scoring algorithm and the category criteria of MDSGene (Klein et al., 2018). The conservation of amino acid sequences was analyzed by using the NCBI BLAST resources (https://blast.ncbi.nlm.nih.gov/Blast.cgi). The locus-specific PCR amplification and the bidirectional Sanger sequencing were performed to confirm variants using the primers 5′-CAATTTAAGGCTAGCTTGAGACTTATG-3′ and 5′-TCTTGAATACTGGGCCACAC-3′, as well as 5′-TCATCATGTTCTTTTTGTGCTTC-3′ and 5′-TGCAAGTTGTCCACGTAATGA-3′. In 100 PD cases, copy number variants (CNVs) were called using the CNVnator (version 0.3.2) read-depth algorithm (Abyzov et al., 2011).

### Statistical Analysis

Fisher's exact test or Pearson's chi-squared test was applied to compare the categorical variables. The Hardy–Weinberg equilibrium for the genotype frequencies of PD subjects and controls was tested. The discrepancy of genotype and allele frequencies between the PD group and the control group was analyzed. Associations between different types of reported *SNCA* gene variants and recorded psychiatric signs as well as cognitive decline/dementia in the literature, which were reported to be common in PD (Book et al., 2018), were assessed. Two-sided *P* < 0.05 were considered statistically significant. The statistical analysis was performed by SPSS statistical software (version 25.0, SPSS Inc., Chicago, IL, USA).

## Results

**Figure 1:**
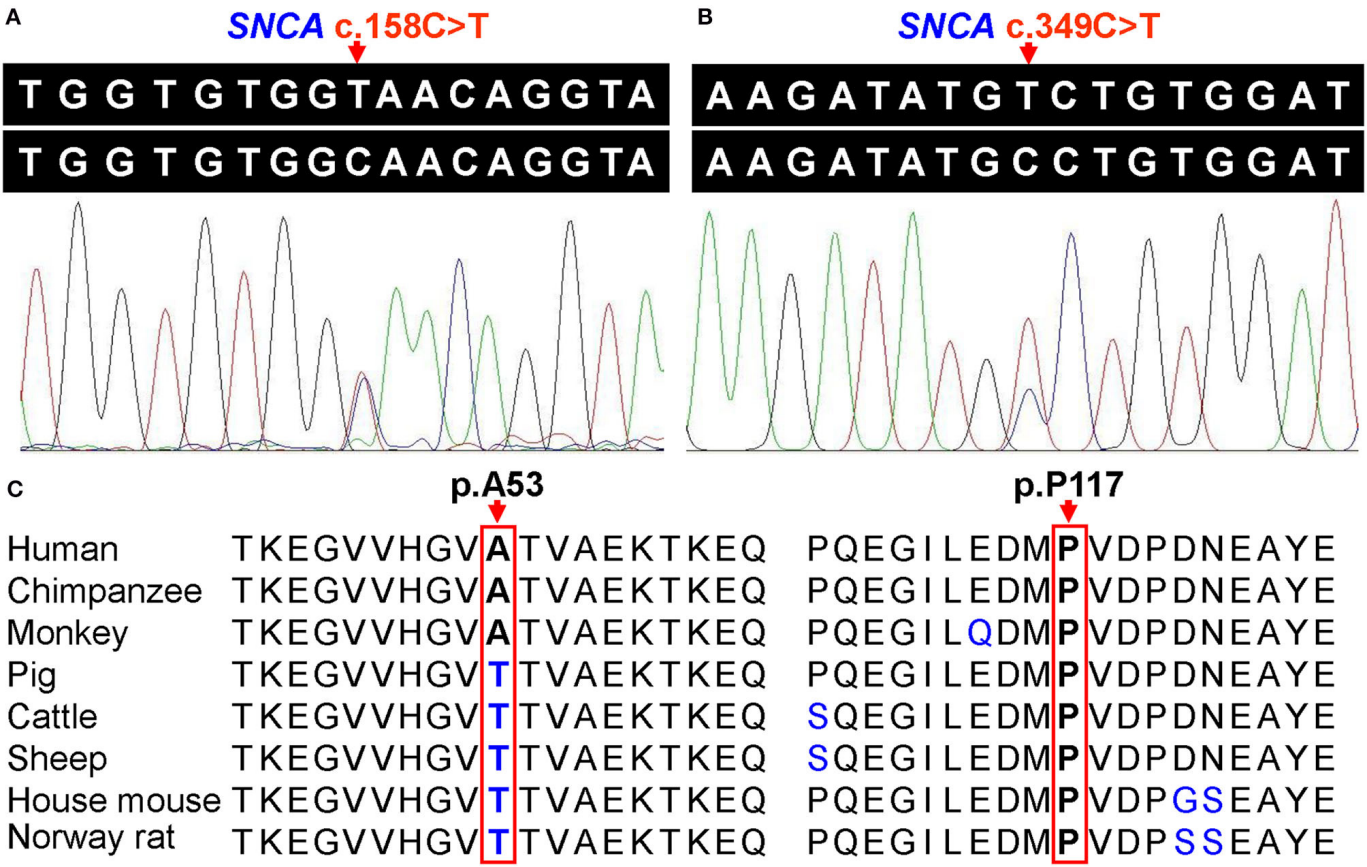
**(A)** Sanger sequencing of heterozygous *SNCA* c.158C>T (p.A53V) variant. **(B)** Sanger sequencing of heterozygous *SNCA* c.349C>T (p.P117S) variant. **(C)** Conservation analysis of alpha-synuclein protein, and arrows indicate the amino acid residues at positions 53 and 117, respectively.

The WES data of the coding regions of the *SNCA* gene and exon–intron boundaries revealed 17 variants, and other putative pathogenic variants for monogenic PD were not detected in these patients. Two known likely pathogenic single-nucleotide nonsynonymous variants (NM_000345.3), namely, c.158C>T (p.A53V, rs542171324) and c.349C>T (p.P117S, rs145138372), were separately detected in two and one patients with PD, respectively (Supplementary Table 1). Patient 1 with p.A53V variant was a 56-year-old female with an onset age of 53 years. The initial symptom was bradykinesia and “pill-rolling” rest tremor in her right upper limb, which subsequently progressed into the left lower limb. The rigidity was also presented on her right limbs. These motor symptoms slowly progressed. Over a period of 3 years, a sleep disorder was manifested. The skull MRI results were normal. Patient 2 with p.A53V variant was a 56-year-old female with an onset age of 52 years. She had similar phenotypic characteristics as those manifested in Patient 1. Patient 3 with p.P117S variant was a 76-year-old male. The initial symptoms were bradykinesia and rest tremor in both upper limbs. He presented typical signs and symptoms of PD such as dystonia in both lower limbs, gait disturbance, and apathy, as well as a sleep disorder. No family history of PD or other related neurodegenerative disease was admitted in these three patients. Both identified variants were confirmed by Sanger sequencing (Figures 1A,B), and neither was observed in any of the 543 controls. SIFT predicted that rs542171324 would be “damaging,” while no potential effects on protein structure or function were revealed by PROVEAN, PolyPhen-2, or MutationTaster. The rs145138372 was predicted to be “disease causing” by MutationTaster, while no potential effects on protein structure or function were revealed by SIFT, PROVEAN, or PolyPhen-2. According to the MDSGene criteria, *SNCA* c.158C>T (p.A53V) and c.349C>T (p.P117S) were interpreted as “probably pathogenic” with a score of 13 and 10, respectively (Table 1). Alanine at position 53 (p.A53) is not conserved between humans and rodents in which threonine is common in most species, while proline at position 117 (p.P117) is conserved in several species (Figure 1C). The Hardy–Weinberg equilibrium testing showed no deviation in either variant (all *P* > 0.05). For both variants (rs542171324 and rs145138372), the case–control study showed no statistically significant differences in genotype or allele frequencies between the PD group and the control group (all *P* > 0.05, Table 2). No CNVs of the *SNCA* gene were detected. Given that the *SNCA* gene mutations have been well confirmed to be responsible for PD worldwide, the *SNCA*-related phenotypes were assessed by extracting 231 cases with parkinsonism as reported in 89 articles (Supplementary Table 2). Psychiatric signs and cognitive decline/dementia were more common among patients with *SNCA* duplication or triplication (psychiatric signs: χ^2^ = 7.892, *P* = 0.005; cognitive decline/dementia: χ^2^ = 8.991, *P* = 0.003, Supplementary Table 3).

**Table 1:** Allelic frequencies and bioinformatics predictions of the *SNCA* rs542171324 and rs145138372 variants.

**dbSNP154**	**Variants**	**Bioinformatics predictions**				**MDSGene**	**Allelic frequencies**			
		**SIFT**	**PolyPhen-2**	**MutationTaster**	**PROVEAN**		**1000G**	**ExAC**	**gnomAD**	**ChinaMap**
rs542171324	c.158C> T (p.A53V)	Damaging	Benign	Polymorphism	Neutral	Probably pathogenic	2 × 10^−4^	8.24 × 10^−6^	7.95 × 10^−6^	1.89 × 10^−4^
rs145138372	c.349C> T (p.P117S)	Tolerated	Benign	Disease causing	Neutral	Probably pathogenic	5.99 × 10^−4^	7.43 × 10^−5^	7.57 × 10^−5^	8.03 × 10^−4^

*SNCA, the alpha-synuclein gene; dbSNP154, Single Nucleotide Polymorphism database version 154; SIFT, Sorting Intolerant from Tolerant; PolyPhen-2, Polymorphism Phenotyping version 2; PROVEAN, Protein Variation Effect Analyzer; MDSGene, Movement Disorder Society Genetic mutation database; 1000G, 1000 Genomes Project; ExAC, Exome Aggregation Consortium; gnomAD, Genome Aggregation Database; ChinaMap, China Metabolic Analytics Project*.

**Table 2:** Genotypic and allelic distributions of rs542171324 and rs145138372 in the Han Chinese patients with PD and controls.

**dbSNP154**	**Genotype/Allele**	**PD**	**Controls**	***P*** **-value**
rs542171324	Genotype			0.199
	CC	436	543	
	CT	2	0	
	TT	0	0	
	Allele			0.199
	C	874	1086	
	T	2	0	
rs145138372	Genotype			0.446
	CC	437	543	
	CT	1	0	
	TT	0	0	
	Allele			0.446
	C	875	1086	
	T	1	0	

*PD, Parkinson's disease; dbSNP154, Single Nucleotide Polymorphism database version 154*.

## Discussion

**Figure 2:**
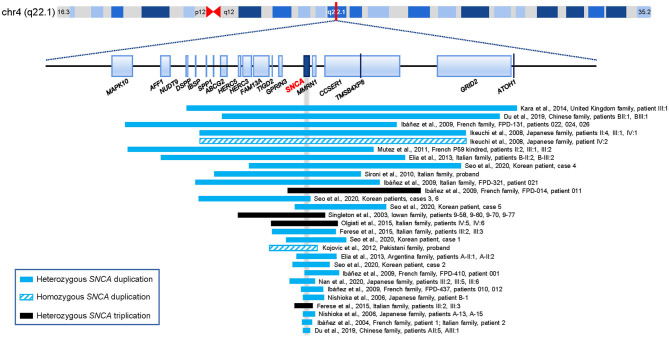
The reported duplications and triplications including the *SNCA* locus for parkinsonism (i.e., bar sizes are not in proportion). The gray vertical bar indicates the position of the *SNCA* gene. The raw data and corresponding references are available in Supplementary Table 2. Genome size is based on human genome build 37.

PD is the most frequent type of synucleinopathy which is pathologically characterized by proteinaceous cytoplasmic inclusions, primarily consisting of alpha-synuclein and ubiquitin (Tagliafierro and Chiba-Falek, 2016; Balestrino and Schapira, 2020; Toffoli et al., 2020). Genetic contributors exert a significant role in the complicated pathogenesis of PD, and considerable progress in establishing its genetic basis has been made since the first disease-causative gene was identified (Hernandez et al., 2016; Deng et al., 2018). Multiple susceptibility genes, disease-causing genes, and genetic risk loci attributed to PD have been identified (Hernandez et al., 2016; Liu and Le, 2020). Intriguingly, some pleomorphic risk loci overlap with the known causative genes of monogenic PD, such as *SNCA*, glucosylceramidase beta (*GBA*), leucine rich repeat kinase 2 (*LRRK2*), and vacuolar protein sorting 13 homolog C (*VPS13C*) (Singleton and Hardy, 2011; Nalls et al., 2019; Blauwendraat et al., 2020). Since the first point mutation of the *SNCA* gene was innovatively identified in 1997, at least 14 missense *SNCA* variants (p.M5T, p.L8I, p.A18T, p.A29S, p.A30P, p.A30G, p.E46K, p.H50Q, p.G51D, p.A53T, p.A53E, p.A53V, p.E57D, and p.P117S) have been discovered to date, which are classified as “pathogenic” or “likely pathogenic” (Polymeropoulos et al., 1997; Youn et al., 2019; Chen et al., 2020; Zhao et al., 2020; Brás et al., 2021; Liu et al., 2021). The CNVs of the *SNCA* locus were discovered in an Iowan kindred for the first time in 2003 (Singleton et al., 2003). Later, *SNCA* genomic multiplications (i.e., duplications and triplications) have been identified in at least 47 families and 18 sporadic cases worldwide (Supplementary Table 2, Figure 2). Apart from point variants and CNVs, some specific short structural variants located in the noncoding regions of the *SNCA* gene, such as promoter Rep1 allele, intron 2 poly-T allele, and intron 4 CT-rich allele, and SNPs, such as rs2736990 and rs356219, were reported to modulate susceptibility to PD (Mata et al., 2010; Miyake et al., 2012; Pan et al., 2013; Guo et al., 2014; Chiba-Falek, 2017; Piper et al., 2018).

The *SNCA* gene, which is mapped to chromosome 4q22.1, consists of six exons, and the latter five exons encode a presynaptic neuronal protein, i.e., alpha-synuclein, with 140 amino acids (Deng and Yuan, 2014; Xu et al., 2015). The protein, a member of the synuclein family, is comprised of an N-terminal with an amphipathic α-helix conformation associated with lipid membrane interactions, a central non-amyloid-component region, and a C-terminal involving fibrillization and aggregation inhibition (Ozansoy and Başak, 2013; Burré, 2015; Xu et al., 2015). Remarkably, almost all identified point variants are located in the N-terminal amphipathic region to date, underlining its significance to the pathogenic mechanism of the alpha-synuclein aggregation (Dehay et al., 2015; Brás et al., 2021). There is a dynamic equilibrium between a natively unfolded monomer and a membrane-bound form of alpha-synuclein protein (Burré, 2015; Dehay et al., 2015). The latter is crucial to mediate the physiological functions implicating synaptic activity and plasticity, neurotransmitter release, dopamine metabolism, synaptic vesicle reserve pool maintenance, and vesicle trafficking at the synapse (Deng and Yuan, 2014; Burré, 2015). The oligomerization or fibrillization of alpha-synuclein by causative point variants and the overexpression of alpha-synuclein by *SNCA* multiplications were proved to have a gain-in-toxic function and gene dosage-related effect, respectively (Tagliafierro and Chiba-Falek, 2016; Stojkovska et al., 2018; Balestrino and Schapira, 2020). The aggregated alpha-synuclein proteins accelerate dopaminergic neuron death and neurodegeneration, which are connected with neurological toxicity pathways, such as the impairment of mitochondrial and synaptic function, the autophagy lysosomal pathway, endoplasmic reticulum overload, and oxidative stress (Emamzadeh, 2017; Fields et al., 2019; Payne et al., 2019).

In this study, two single-nucleotide variants of the *SNCA* gene, namely, c.158C>T (p.A53V, rs542171324) and c.349C>T (p.P117S, rs145138372), were identified in three patients with PD. The rs542171324 has been previously reported in a Japanese autosomal dominant PD family in the homozygous state and three unrelated Chinese patients with early-onset PD (Yoshino et al., 2017; Chen et al., 2020). The rs145138372 has been found in a single patient of European ancestry with isolated/idiopathic rapid eye movement sleep behavior disorder and a Chinese patient with familial PD (Krohn et al., 2020; Zhao et al., 2020).

Considering p.A53V and p.P117S observed in sporadic PD cases and unaffected individuals of small families, as well as obtained weak bioinformatics evidence of pathogenicity and the relatively high frequency in Chinese patients, we inferred that these two variants may exert a high probability of susceptibility in PD rather than act as pathogenic variants in monogenic PD. To evaluate the relationship between *SNCA* gene variants and PD in the Han Chinese population, a well-characterized case–control comparison study was conducted to further investigate the relevance of two SNPs in the PD susceptibility in the Han Chinese individuals from mainland China. Despite the immense interest and considerable effort, no significant association between the two SNPs and the development of PD was detected (all *P* > 0.05). This study indicates that these SNPs in the coding regions of the *SNCA* gene have less or no effect on the development of PD in the Han Chinese population. No CNVs of the *SNCA* gene were detected.

Interestingly, an *in vitro* study showed that *SNCA* c.158C>T (p.A53V) variant increases the aggregation propensity and promotes oligomerization and fibrillation that are known to be related to the pathogenesis of PD (Mohite et al., 2018). It is also worth noting that the *SNCA* c.158C>T (p.A53V) variant, which is classified as “pathogenic,” was detected in three unrelated Chinese patients sharing the same haplotype (Chen et al., 2020). The *SNCA* c.349C>T (p.P117S) variant identified in the Chinese patients was classified as “likely pathogenic,” and its pathogenicity was supported by its effect on protein solubility (Zhao et al., 2020). However, the changes of *in vitro* functional experiments are not found to be enough to prove that those two variants may bring the carriers to reach the threshold of the development of PD. Recruitment of more families for detecting the variants and further functional analyses like *in vivo* experimental studies are warranted for their definite roles in PD.

Patients with PD, particularly those with *SNCA* gene multiplication, are usually complicated with neuropsychiatric symptoms, such as visual hallucination, delusion, and cognitive decline/dementia (Book et al., 2018). We reviewed all reported cases with *SNCA* variants worldwide. Psychiatric signs and cognitive decline/dementia were more common in patients with *SNCA* multiplications. This study may be limited by the applied methods for not fully analyzing the variants in the noncoding region of the *SNCA* gene.

## Conclusion

The two variants, namely, c.158C>T (p.A53V, rs542171324) and c.349C>T (p.P117S, rs145138372), of the *SNCA* gene were detected. The study shows that variants located in the coding regions of the *SNCA* gene may have less or no role in the development of PD in the Han Chinese population. Associations between different variant types of the *SNCA* gene and psychiatric signs as well as cognitive decline/dementia were assessed, which were found to be more common among patients with *SNCA* duplication or triplication.

## Data Availability Statement

The raw data presented in this article will be available by the authors, and reasonable requests to access the datasets by the qualified researchers should be directed to the corresponding author after legal permission.

## Ethics Statement

The studies involving human participants were reviewed and approved by the Institutional Review Board of the Third Xiangya Hospital of Central South University. The patients/participants provided their written informed consent to participate in this study.

## Author Contributions

LY and HD contributed to the conception and design of the study. ZS, WZ, WX, and YY performed patient samples and clinical data acquisition. YG, YS, and YY performed the experiments. YG, YS, LY, and HD were involved in the data analysis and wrote the manuscript. All authors read and approved the final version of the manuscript.

## Conflict of Interest

The authors declare that the research was conducted in the absence of any commercial or financial relationships that could be construed as a potential conflict of interest.

## Publisher's Note

All claims expressed in this article are solely those of the authors and do not necessarily represent those of their affiliated organizations, or those of the publisher, the editors and the reviewers. Any product that may be evaluated in this article, or claim that may be made by its manufacturer, is not guaranteed or endorsed by the publisher.
