# Supplementary material for: Genetic Analysis and Literature Review of SNCA Variants in Parkinson's Disease
Source: Front Aging Neurosci. 2021 Aug 12;13:648151. doi: 10.3389/fnagi.2021.648151 (PMC8397385; doi:10.3389/fnagi.2021.648151)
Supplement: Supplementary file 1 [file Data_Sheet_1.pdf]

Supplementary Table 1. The detected variants of the *SNCA* gene by whole exome sequencing.

No.	Position (hg19)	dbSNP154 ID	Variants [a]	Allele frequencies					
				1000G	ExAC	gnomAD	ChinaMap	Case [b]	Control [b]
1	chr4: 90647992	-	c.391-181C>G	-	-	-	$9.44×10^{-5}$	0	$9.21×10^{-4}$
2	chr4: 90648042	rs554432760	c.391-231A>T	$2×10^{-4}$	-	$6.4×10^{-5}$	$6.14×10^{-4}$	0	$9.21×10^{-4}$
3	chr4: 90650172	rs34511254	c.390+172dupA	-	-	-	-	$2.17×10^{-2}$	$1.10×10^{-2}$
4	chr4: 90650227	-	c.390+118A>T	-	-	-	-	0	$3.68×10^{-3}$
5	chr4: 90650254	rs77010743	c.390+91T>G	$1.92×10^{-2}$	-	$5.07×10^{-3}$	$8.73×10^{-2}$	0	$9.21×10^{-4}$
6	chr4: 90650309	rs144274752	c.390+36T>C	$2.20×10^{-3}$	$3.39×10^{-3}$	$3.80×10^{-3}$	$4.82×10^{-3}$	$6.85×10^{-3}$	$5.52×10^{-3}$
7	chr4: 90650354	rs191055637	c.381G>A (p.Met127Ile)	$2×10^{-4}$	$9.9×10^{-5}$	$7.44×10^{-5}$	$4.25×10^{-4}$	0	$9.21×10^{-4}$
**8**	**chr4: 90650386**	**rs145138372**	**c.349C>T (p.Pro117Ser)**	**$5.99×10^{-4}$**	**$7.43×10^{-5}$**	**$7.57×10^{-5}$**	**$8.03×10^{-4}$**	**$1.14×10^{-3}$**	**0**
9	chr4: 90650544	-	c.307-116A>C	-	-	-	$4.72×10^{-5}$	$1.14×10^{-3}$	$9.21×10^{-4}$
10	chr4: 90650613	-	c.307-185C>T	-	-	-	$1.37×10^{-3}$	$1.14×10^{-3}$	0
11	chr4: 90743555	-	c.164-16T>C	-	-	-	-	0	$9.21×10^{-4}$
12	chr4: 90743821	rs191701032	c.164-282C>T	$3.99×10^{-4}$	-	$3.5×10^{-4}$	$4.96×10^{-3}$	$2.28×10^{-3}$	$9.21×10^{-4}$
13	chr4: 90749136	-	c.163+158T>A	-	-	-	$4.72×10^{-5}$	$2.28×10^{-3}$	0
**14**	**chr4: 90749299**	**rs542171324**	**c.158C>T (p.Ala53Val)**	**$2×10^{-4}$**	**$8.24×10^{-6}$**	**$7.95×10^{-6}$**	**$1.89×10^{-4}$**	**$2.28×10^{-3}$**	**0**
15	chr4: 90749368	rs34304288	c.122-33C>T	$7.39×10^{-3}$	$2.76×10^{-3}$	$2.90×10^{-3}$	$3.67×10^{-2}$	0	$9.21×10^{-4}$
16	chr4: 90756445	-	c.121+253G>T	-	-	-	$4.72×10^{-4}$	$1.14×10^{-3}$	0
17	chr4: 90756550	rs7681440	c.121+148G>C	-	-	-	0.88	0.90	0.87

SNCA, the alpha-synuclein gene; dbSNP154, Single Nucleotide Polymorphism database version 154; 1000G, 1000 Genomes Project; ExAC, Exome Aggregation Consortium; gnomAD, Genome Aggregation Database; ChinaMap, China Metabolic Analytics Project; -, no data available.

[a] *Nomenclature for the identified variants is made in accordance with the recommendations of the Human Genome Variation Society (https://varnomen.hgvs.org/) using the reference sequence NM_000345.3.*

[b] *In this study.*

Supplementary Table 2. Summary of clinical and genetic features of patients with the *SNCA* gene variants.

Origin/Ethnicity	Enrolled cases	Family history	OA (age range, years)	Variants [a]	Clinical features	References
Chinese	1	No	45	M5T [b]	B, R, T, PI, gait disturbance	Zhao et al., 2020
Chinese	1	No	37.6	L8I [b]	Typical PD signs, CD, L-dopa (+)	Chen et al., 2020
Polish	1	No	50	A18T [b]	B, R, T, PI, autonomic dysfunction, dementia, L-dopa (+)	Hoffman-Zacharska et al., 2013
Polish	1	No	60	A29S [b]	B, R, T, PI, psychiatric signs (depression, anxiety), L-dopa (+)	Hoffman-Zacharska et al., 2013
German	2	Yes	NA	A30P	B, R, T, gait disturbance, hypomimia, hypophonia, L-dopa (+)	Krüger et al., 1998
German	3	Yes	61.7 (54-76)	A30P	B, R, T, PI, gait disturbance, hypomimia, hypophonia, psychiatric sign (visual hallucination), CD, L-dopa (+)	Krüger et al., 2001
Greek	5	Yes	50.8 (36-60)	A30G [b]	B, R, T, PI, gait disturbance, psychiatric signs (hallucination, depression, delusion, anxiety, apathy, disinhibition), genitourinary dysfunction, RBD, OH, CD/dementia, L-dopa (+)	Liu et al., 2021
Spanish	9	Yes	55 (28-67)	E46K	B, R, T, PI, gait disturbance, hypophonia, hyposmia, psychiatric signs (visual hallucination, delusion, depression, confusion), RBD, OH, CD/dementia, L-dopa (+)	Zarranz et al., 2004, 2005; Somme et al., 2011; Tijero et al., 2013
Bolivian	3	Yes	53.7 (50-58)	E46K	B, R, T, hyposmia, psychiatric signs (depression, anxiety), gastrointestinal dysfunction, genitourinary	Pimentel et al., 2015

Origin/Ethnicity	Enrolled cases	Family history	OA (age range, years)	Variants [a]	Clinical features	References
					dysfunction, RBD	
Caucasian English	1	No	71	H50Q	T, CD, L-dopa (+)	Proukakis et al., 2013
English/Welsh	1	Yes	60	H50Q	B, R, T, hypomimia, micrographia, hypophonia, psychiatric signs (anxiety, apathy), dementia, L-dopa (+)	Appel-Cresswell et al., 2013
French	3	Yes	42 (31-60)	G51D	B, R, T, psychiatric signs (hallucination, delusion, depression, anxiety), genitourinary dysfunction, spasticity, pyramidal signs (enhanced tendon reflexes, bilateral extensor plantar reflexes), L-dopa (+)	Lesage et al., 2013
British	2	Yes	29.5 (19-40)	G51D	B, R, T, PI, dysarthria, stiffness, psychiatric sign (visual hallucination), OH, CD, myoclonus, bilateral extensor plantar response, L-dopa (+)	Kiely et al., 2013
Japanese	1	Yes	28	G51D	B, R, PI, gait disturbance, psychiatric signs (visual hallucination, delusion, impulsion, excitability), genitourinary dysfunction, OH, dementia, myoclonus, hyperreflexia, L-dopa (+)	Tokutake et al., 2014
British Caucasian	2	Yes	57.5 (46-69)	G51D	B, R, T, gait disturbance, hypomimia, micrographia, dysphagia, psychiatric signs (visual hallucination, delusion, depression, anxiety, confusion), genitourinary dysfunction, OH,	Kiely et al., 2015

Origin/Ethnicity	Enrolled cases	Family history	OA (age range, years)	Variants [a]	Clinical features	References
					CD/dementia, hyperreflexia, L-dopa (+)	
Italian	6	Yes	46±13	A53T	B, R, PI, stiffness, gait disturbance, psychiatric sign (depression), dementia, L-dopa (+)	Golbe et al., 1990; Polymeropoulos et al., 1997
Greek	4	Yes	30s-50s	A53T	NA	Polymeropoulos et al., 1997
Greek	10	Yes	48.4 (36-58)	A53T	B, R, T	Polymeropoulos et al., 1997; Athanassiadou et al., 1999
Greek	4	Yes	43 (39-49)	A53T	B, R, T, PI, gait disturbance, hypomimia, hypophonia, L-dopa (+)	Papadimitriou et al., 1999
Greek-American	5	Yes	50.2 (31-61)	A53T	NA	Scott et al., 1999
Greek-Australian	3	Yes	44.3 (42-46)	A53T	B, R, T, gait disturbance, dysphagia, psychiatric sign (hallucination, apathy, confusion), genitourinary dysfunction, OH, CD, myoclonus, L-dopa (+)	Spira et al., 2001
Greek	15	Yes	47.9 (25-64)	A53T	B, R, T, PI, psychiatric sign (depression), OH, CD	Papapetropoulos et al., 2001
Greek	8	Yes	39.8 (32-50)	A53T	B, R, T, masked face, dysarthria, hyposmia, psychiatric sign (depression), CD, L-dopa (+)	Bostantjopoulou et al., 2001
Polish	1	No	74	A53T	B, R, T, gait disturbance, L-dopa (+)	Michell et al., 2005
Greek	1	Yes	NA	A53T	Akinetic-rigid type, L-dopa (+)	Berg et al., 2005
Greek	1	Yes	67	A53T	B, R, T, dysphagia, psychiatric sign (visual hallucination), genitourinary dysfunction, OH, dementia	Morfis and Cordato, 2006

Origin/Ethnicity	Enrolled cases	Family history	OA (age range, years)	Variants [a]	Clinical features	References
Korean	1	Yes	35	A53T	B, R, stiffness, hypomimia, hypophonia, L-dopa (+)	Ki et al., 2007
Greek-American	8	Yes	48.6 (31-59)	A53T	B, R, T, PI, sleep disturbance, OH, dementia, myoclonus, L-dopa (+)	Markopoulou et al., 1995, 1999, 2008
Swedish	1	Yes	39	A53T	B, R, T, gait disturbance, stiffness, hypomimia, dysarthria, psychiatric sign, genitourinary dysfunction, myoclonus, dementia, L-dopa (+)	Puschmann et al., 2009
Greek	5	Yes	43.6 (31-61)	A53T	NA	Bozi et al., 2014
Italian	4	Yes	32.7±10.5 (26-48)	A53T	B, R, T, PI, hyposmia, psychiatric signs (depression, anxiety), dysautonomia, CD, L-dopa (+)	Ricciardi et al., 2016
Chinese	1	No	22	A53T	B, R, T, PI, stiffness, hypomimia, hyposmia, L-dopa (+)	Xiong et al., 2016
Italian	1	Yes	58	A53T	B, stiffness, hyposmia, psychiatric signs (sadness, loss of initiative), RBD, OH	Tambasco et al., 2016
Greek	2	Yes	39.5 (30-49)	A53T	B, R, T, PI, gait disturbance, hypomimia, hypophonia, psychiatric signs (anxiety, apathy), genitourinary dysfunction, CD, myoclonus, hyperreflexia, L-dopa (+)	Bougea et al., 2017
Greek	4	Yes	36.8 (30-44)	A53T	Parkinsonism, psychiatric signs (visual hallucination, paranoid ideation, apathy, executive dysfunction, fluctuating attentional	Breza et al., 2018

Origin/Ethnicity	Enrolled cases	Family history	OA (age range, years)	Variants [a]	Clinical features	References
					deficits, impaired visuospatial skills), dementia	
Finnish	3	Yes	43.3 (32-62)	A53E	Parkinsonism, psychiatric signs (anxiety, panic), insomnia, OH, spasticity, myoclonic jerk, L-dopa (+)	Pasanen et al., 2014
Finnish	2	Yes	33.5 (25-42)	A53E	B, R, T, gait disturbance, hypomimia, dysarthria, gastrointestinal dysfunction, L-dopa (+)	Martikainen et al., 2015
Finnish	1	Yes	41	A53E	Typical PD signs, dysarthria, dysphagia	Pasanen et al., 2017
Japanese	1	Yes	55	A53V [b] (hom)	B, R, T, psychiatric sign (visual hallucination), RBD, dementia, L-dopa (+)	Yoshino et al., 2017
Chinese	3	Yes (1/3)	37.1 (35.81-39.10)	A53V [b]	Typical PD signs, hyposmia, psychiatric sign (depression), RBD, CD, L-dopa (+)	Chen et al., 2020
Korean	1	No	48	E57D [b]	Dystonia, hyposmia, gastrointestinal dysfunction	Youn et al., 2019
Chinese	1	Yes	50	P117S [b]	B, R, T, PI, gait disturbance	Zhao et al., 2020
Korean	1	No	44	c.*464C>A	Typical PD, L-dopa (+)	Kim et al., 2013
French	4	Yes	50.8 (40-65)	Dup (4.928 Mb)	B, R, T, PI, dementia, L-dopa (+)	Chartier-Harlin et al., 2004; Mutez et al., 2011
French, Italian	2	Yes	48 (46-50)	Dup (>204 Kb, >206 Kb)	B, R, T, psychiatric sign (depression), L-dopa (+)	Ibáñez et al., 2004
Japanese	2	Yes	43 (38-48)	Dup (220	B, R, PI, gait disturbance, psychiatric	Nishioka et al., 2006

Origin/Ethnicity	Enrolled cases	Family history	OA (age range, years)	Variants [a]	Clinical features	References
				Kb)	signs (depression, psychosis), L-dopa (+)	
Japanese	1	Yes	47	Dup (394 Kb)	B, R, gait disturbance, psychiatric sign (hallucination), CD, L-dopa (+)	Nishioka et al., 2006
Swedish	1	Yes	71	Dup (<0.9 Mb)	B, R, T, PI, psychiatric signs (hallucination, paranoia, depression, anxiety), genitourinary dysfunction, OH, dementia, myoclonus, L-dopa (+)	Fuchs et al., 2007
Korean	6	Yes (1/6)	52 (40-66)	Dup (718.3 -4162 Kb)	B, R, T, PI, gait disturbance, dysphonia, psychiatric signs (visual hallucination, depression, anxiety), gastrointestinal dysfunction, RBD, OH, CD, L-dopa (+)	Ahn et al., 2008; Shin et al., 2010; Seo et al., 2020
Japanese	4	Yes	49.8 (28-71)	Dup (5 Mb, 1 hom/3 het)	B, R, T, PI, gait disturbance, masked face, hypophonia, psychiatric signs (visual hallucination, delusion, attention impairment), gastrointestinal dysfunction, genitourinary dysfunction, dementia, OH, myoclonus, L-dopa (+)	Ishikawa et al., 1997; Ikeuchi et al., 2008
Japanese	2	Yes	55 (42-68)	Dup (0.5-1.6 Mb)	B, R, T, gait disturbance, psychiatric signs (visual hallucination, depression, anxiety), CD, L-dopa (+)	Uchiyama et al., 2008
German	1	No	36	Dup	B, R, PI, hyposmia, L-dopa (+)	Brueggemann et al., 2008

Origin/Ethnicity	Enrolled cases	Family history	OA (age range, years)	Variants [a]	Clinical features	References
NA	1	No	35	Dup	B, T, OH, L-dopa (+)	Troiano et al., 2008
French, Italian	7	Yes	44.3 (38-50)	Dup [c]	B, R, T, L-dopa (+)	Ibáñez et al., 2009
Belgian	1	NA	68	Dup	R, T, CD, L-dopa (+)	Nuytemans et al., 2009
Japanese	7	Yes	49.9 (37-62)	Dup	B, R, T, PI, gait disturbance, hyposmia, dysphagia, psychiatric signs (hallucination, depression, delusion), RBD, dementia, L-dopa (+)	Nishioka et al., 2009
Japanese	1	No	31	Dup	B, gait disturbance, hyposmia, psychiatric signs (hallucination, depression, delusion), RBD, L-dopa (+)	Nishioka et al., 2009
Italian	1	Yes	41	Dup (3.65 Mb)	B, R, psychiatric signs (depression, anxiety, panic attack), genitourinary dysfunction, OH, L-dopa (+)	Sironi et al., 2010
White	1	No	30	Dup (41.2 Mb)	B, R, T, gait disturbance, genitourinary dysfunction, mental retardation, L-dopa (+)	Garraux et al., 2012
Belgian	1	No	77	Dup	R, psychiatric signs (visual hallucination), CD	Meeus et al., 2012:
Pakistani	1	No	31	Dup (0.928 Mb, hom)	B, R, T, stiffness, gait disturbance, micrographia, psychiatric signs (depression, psychosis), CD	Kojovic et al., 2012
Asian	1	Yes	20s	Dup	B, R, T, gait disturbance, attentiveness decline, dystonia, L-dopa (+)	Itokawa et al., 2013

**Origin/Ethnicity**	**Enrolled cases**	**Family history**	**OA (age range, years)**	**Variants [a]**	**Clinical features**	**References**
Northern Argentina	2	Yes	43.5 (43-44)	Dup (773 Kb)	T, dysphagia, psychiatric signs (visual and acoustic hallucinations, depression), gastrointestinal dysfunction, genitourinary dysfunction, RBD, OH, dementia, L-dopa (+)	Elia et al., 2013
Italian	2	Yes	55 (32-78)	Dup (4820 Kb)	B, R, T, PI, hypomimia, psychiatric signs (visual and acoustic hallucinations, depression, delusion, physical aggressiveness), CD, L-dopa (+)	Elia et al., 2013
Non-Hispanic Caucasian	1	Yes	36	Dup	NA	Wang et al., 2013
Iranian	5	Yes (4/5)	40.8	Dup	Typical PD	Darvish et al., 2013
United Kingdom	1	Yes	38	Dup (6.4 Mb)	R, T, akinetic-rigid syndrome, dysarthria, psychiatric signs (hallucination, anxiety, panic disorder), RBD, CD, L-dopa (+)	Kara et al., 2014
Italian	2	Yes	35 (28-42)	Dup (1.29 Mb)	Hypokinetic-rigid syndrome, dysarthria, dysphagia, psychiatric signs (visual hallucination, depression, delusion, jealousy, aggressive behaviors), genitourinary dysfunction, RBD, CD	Ferese et al., 2015

Origin/Ethnicity	Enrolled cases	Family history	OA (age range, years)	Variants [a]	Clinical features	References
American	1	No	48	Dup	B, R, T, PI, psychiatric signs (visual hallucination, depression, confusion), gastrointestinal dysfunction, genitourinary dysfunction, RBD, OH, dementia	Konno et al., 2016
Japanese	1	Yes	43	Dup	B, R, PI, gait disturbance, masked face, psychiatric signs (auditory hallucination, schizophrenia)	Takamura et al., 2016
European-American	1	Yes	67	Dup	Idiopathic PD	Benitez et al., 2016
Turkish	2	Yes	43.5 (41-46)	Dup	Parkinsonism, dementia	Kessler et al., 2018
Hungarian	1	No	NA	Dup	NA	Illés et al., 2019
Swedish	1	Yes	52	Dup	B, R, CD, RBD	Puschmann et al., 2019
Chinese	2	Yes	55.5 (50-61)	Dup (139 Kb)	B, R, T, PI, hyposmia, psychiatric signs (visual hallucination, depression), RBD, dementia, L-dopa (+)	Du et al., 2019

**Origin/Ethnicity**	**Enrolled cases**	**Family history**	**OA (age range, years)**	**Variants [a]**	**Clinical features**	**References**
Chinese	2	Yes	53.5 (38-69)	Dup (5.4 Mb)	B, R, T, PI, hyposmia, psychiatric sign (depression), gastrointestinal dysfunction, RBD, CD, L-dopa (+)	Du et al., 2019
Chinese	3	Yes	39.7 (34-46)	Dup	B, R, T, PI, gait disturbance, hyposmia, psychiatric sign (depression), RBD, CD	Zhao et al., 2020
Hispanic American	1	Yes	NA	Dup (248 Kb)	R, T, gait disturbance, clonus, hyperreflexia	Robak et al., 2020
Japanese	3	Yes	49.7 (43-62)	Dup (476.5 Kb)	B, R, T, PI, gait disturbance, masked face, hoarseness, psychiatric signs (hallucination, depression), gastrointestinal dysfunction, OH, L-dopa (+)	Nan et al., 2020
Iowan	5	Yes	34.8 (24-46)	Trip (1.7 Mb)	B, R, T, PI, psychiatric signs (hallucination, depression), gastrointestinal dysfunction, genitourinary dysfunction, RBD, OH, dementia, myoclonus, L-dopa (+)	Muenter et al., 1998; Singleton et al., 2003; Ross et al., 2008; Zafar et al., 2018
Swedish-American	1	Yes	31	Trip (<0.9 Mb)	B, R, T, PI, dysphagia, psychiatric signs (visual, auditory and olfactory hallucinations, paranoia, depression, anxiety), genitourinary dysfunction, OH, dementia, L-dopa (+)	Farrer et al., 2004; Fuchs et al., 2007
Iowan	2	Yes	NA	Trip	B, R, T, PI, gait disturbance, micrographia, hypophonia, autonomic signs, L-dopa (+)	Singleton et al., 2004

Origin/Ethnicity	Enrolled cases	Family history	OA (age range, years)	Variants [a]	Clinical features	References
French	1	Yes	48	Trip (2.61-2.64 Mb)	B, R, T, genitourinary dysfunction, CD, L-dopa (+)	Ibáñez et al., 2009
French-Italian	1	Yes	46	Trip	Typical PD, psychiatric sign (psychosis), autonomic signs, dementia, L-dopa (+)	Keyser et al., 2010
Japanese	1	Yes	28	Trip	B, R, T, gait disturbance, masked face, OH, L-dopa (+)	Sekine et al., 2010
Iranian	4	Yes (3/4)	25.3	Trip	Typical PD, dementia	Darvish et al., 2013
Italian	2	Yes	35 (28-42)	Trip (351 Kb)	Hypokinetic-rigid syndrome, dysarthria, dysphagia, psychiatric signs (visual hallucination, depression, delusion, jealousy, aggressive behavior), genitourinary dysfunction, RBD, CD	Ferese et al., 2015
Italian	2	Yes	35 (28-42)	Trip (1.29-1.3 Mb)	B, R, T, hypomimia, psychiatric signs (depression, delirium of jealousy, aggressive behavior), genitourinary dysfunction, RBD, CD, L-dopa (+)	Olgiati et al., 2015
Korean	2	No	44.5 (44-45)	Trip	Dystonia, psychiatric sign (depression), gastrointestinal dysfunction, CD	Youn et al., 2019

SNCA, the alpha-synuclein gene; OA, mean onset age (years); PD, Parkinson's disease; B, bradykinesia; R, rigidity; T, resting tremor; PI, postural instability; RBD, rapid eye movement sleep behavior disorder; OH, orthostatic hypotension; CD, cognitive decline; L-dopa (+), a positive response to levodopa therapy; NA, no data available; Dup, duplication; Trip, triplication; Kb, kilobases; Mb, megabases; hom,

homozygote; het, heterozygote.
[a] *Definitely pathogenic variants in Movement Disorder Society Genetic mutation database (MDSGene): p.A30P, p.G51D, p.A53T, p.A53E, duplication and triplication; probably pathogenic variants in MDSGene: p.E46K and c.*464C>A; possibly pathogenic variant in MDSGene: p.H50Q.*
[b] *Reported variants not recorded in MDSGene.*
[c] *4.50-5.29 Mb duplication in FPD-131, 3.47-3.58 Mb duplication in FPD-321, 0.63-0.65 Mb duplication in FPD-410, and 0.42-0.43 Mb duplication in FPD-437.*

REFERENCES

Ahn, T. B., Kim, S. Y., Kim, J. Y., Park, S. S., Lee, D. S., Min, H. J., et al. (2008). alpha-Synuclein gene duplication is present in sporadic Parkinson disease. *Neurology* 70, 43-49. doi: 10.1212/01.wnl.0000271080.53272.c7.

Appel-Cresswell, S., Vilarino-Guell, C., Encarnacion, M., Sherman, H., Yu, I., Shah, B., et al. (2013). Alpha-synuclein p.H50Q, a novel pathogenic mutation for Parkinson's disease. *Mov. Disord.* 28, 811-813. doi: 10.1002/mds.25421.

Athanassiadou, A., Voutsinas, G., Psiouri, L., Leroy, E., Polymeropoulos, M. H., Ilias, A., et al. (1999). Genetic analysis of families with Parkinson disease that carry the Ala53Thr mutation in the gene encoding alpha-synuclein. *Am. J. Hum. Genet.* 65, 555-558. doi: 10.1086/302486.

Benitez, B. A., Davis, A. A., Jin, S. C., Ibanez, L., Ortega-Cubero, S., Pastor, P., et al. (2016). Resequencing analysis of five Mendelian genes and the top genes from genome-wide association studies in Parkinson's Disease. *Mol. Neurodegener.* 11, 29. doi: 10.1186/s13024-016-0097-0.

Berg, D., Niwar, M., Maass, S., Zimprich, A., Möller, J. C., Wuellner, U., et al. (2005). Alpha-synuclein and Parkinson's disease: implications from the screening of more than 1,900 patients. *Mov. Disord.* 20, 1191-1194. doi: 10.1002/mds.20504.

Bostantjopoulou, S., Katsarou, Z., Papadimitriou, A., Veletza, V., Hatzigeorgiou, G., and Lees, A. (2001). Clinical features of parkinsonian patients with the alpha-synuclein (G209A) mutation. *Mov. Disord.* 16, 1007-1013. doi: 10.1002/mds.1221.

Bougea, A., Koros, C., Stamelou, M., Simitsi, A., Papagiannakis, N., Antonelou, R., et al. (2017). Frontotemporal dementia as the presenting phenotype of p.A53T mutation carriers in the alpha-synuclein gene. *Parkinsonism Relat. Disord.* 35, 82-87. doi: 10.1016/j.parkreldis.2016.12.002.

Bozi, M., Papadimitriou, D., Antonellou, R., Moraitou, M., Maniati, M., Vassilatis, D. K., et al. (2014). Genetic assessment of familial and early-onset Parkinson's disease in a Greek population. *Eur. J. Neurol.* 21, 963-968. doi: 10.1111/ene.12315.

Breza, M., Koutsis, G., Karadima, G., Potagas, C., Kartanou, C., Papageorgiou, S. G., et al. (2018). The different faces of the p. A53T alpha-synuclein mutation: a screening of Greek patients with parkinsonism and/or dementia. *Neurosci. Lett.* 672, 136-139. doi:

10.1016/j.neulet.2017.12.015.
Brueggemann, N., Odin, P., Gruenewald, A., Tadic, V., Hagenah, J., Seidel, G., et al. (2008). Re: Alpha-synuclein gene duplication is present in sporadic Parkinson disease. *Neurology* 71, 1294. doi: 10.1212/01.wnl.0000338439.00992.c7.
Chartier-Harlin, M. C., Kachergus, J., Roumier, C., Mouroux, V., Douay, X., Lincoln, S., et al. (2004). Alpha-synuclein locus duplication as a cause of familial Parkinson's disease. *Lancet* 364, 1167-1169. doi: 10.1016/S0140-6736(04)17103-1.
Chen, Y., Gu, X., Ou, R., Zhang, L., Hou, Y., Liu, K., et al. (2020). Evaluating the role of SNCA, LRRK2, and GBA in Chinese patients with early-onset Parkinson's disease. *Mov. Disord.* 35, 2046-2055. doi: 10.1002/mds.28191.
Darvish, H., Movafagh, A., Omrani, M. D., Firouzabadi, S. G., Azargashb, E., Jamshidi, J., et al. (2013). Detection of copy number changes in genes associated with Parkinson's disease in Iranian patients. *Neurosci. Lett.* 551, 75-78. doi: 10.1016/j.neulet.2013.07.013.
Du, Y. J., Shen, Y., Wang, Y. X., Sun, Y. M., Liu, F. T., Chen, C., et al. (2019). Clinical variability in Chinese families with Parkinson disease and SNCA duplication, including the shortest 139kb duplication. *Parkinsonism Relat. Disord.* 68, 60-62. doi: 10.1016/j.parkreldis.2019.09.030.
Elia, A. E., Petrucci, S., Fasano, A., Guidi, M., Valbonesi, S., Bernardini, L., et al. (2013). Alpha-synuclein gene duplication: marked intrafamilial variability in two novel pedigrees. *Mov. Disord.* 28, 813-817. doi: 10.1002/mds.25518.
Farrer, M., Kachergus, J., Forno, L., Lincoln, S., Wang, D. S., Hulihan, M., et al. (2004). Comparison of kindreds with parkinsonism and alpha-synuclein genomic multiplications. *Ann. Neurol.* 55, 174-179. doi: 10.1002/ana.10846.
Ferese, R., Modugno, N., Campopiano, R., Santilli, M., Zampatti, S., Giardina, E., et al. (2015). Four copies of SNCA responsible for autosomal dominant Parkinson's disease in two Italian siblings. *Parkinsons Dis.* 2015, 546462. doi: 10.1155/2015/546462.
Fuchs, J., Nilsson, C., Kachergus, J., Munz, M., Larsson, E. M., Schüle, B., et al. (2007). Phenotypic variation in a large Swedish pedigree due to SNCA duplication and triplication. *Neurology* 68, 916-922. doi: 10.1212/01.wnl.0000254458.17630.c5.
Garraux, G., Caberg, J. H., Vanbellinghen, J. F., Jamar, M., Bours, V., Moonen, G., et al. (2012). Partial trisomy 4q associated with young-onset dopa-responsive parkinsonism. *Arch. Neurol.* 69, 398-400. doi: 10.1001/archneurol.2011.802.
Golbe, L. I., Di Iorio, G., Bonavita, V., Miller, D. C., and Duvoisin, R. C. (1990). A large kindred with autosomal dominant Parkinson's disease. *Ann. Neurol.* 27, 276-282. doi: 10.1002/ana.410270309.
Hoffman-Zacharska, D., Koziorowski, D., Ross, O. A., Milewski, M., Poznanski, J. A., Jurek, M., et al. (2013). Novel A18T and pA29S substitutions in α-synuclein may be associated with sporadic Parkinson's disease. *Parkinsonism Relat. Disord.* 19, 1057-1060. doi: 10.1016/j.parkreldis.2013.07.011.
Ibáñez, P., Lesage, S., Janin, S., Lohmann, E., Durif, F., Destée, A., et al. (2009). Alpha-synuclein gene rearrangements in dominantly inherited parkinsonism: frequency, phenotype, and mechanisms. *Arch. Neurol.* 66, 102-108. doi: 10.1001/archneurol.2008.555.
Ibáñez, P., Bonnet, A. M., Débarges, B., Lohmann, E., Tison, F., Pollak, P., et al. (2004). Causal relation between alpha-synuclein gene duplication and familial Parkinson's disease. *Lancet* 364, 1169-1171. doi: 10.1016/S0140-6736(04)17104-3.

Ikeuchi, T., Kakita, A., Shiga, A., Kasuga, K., Kaneko, H., Tan, C. F., et al. (2008). Patients homozygous and heterozygous for SNCA duplication in a family with parkinsonism and dementia. *Arch. Neurol.* 65, 514-519. doi: 10.1001/archneur.65.4.514.

Illés, A., Csabán, D., Grosz, Z., Balicza, P., Gézsi, A., Molnár, V., et al. (2019). The role of genetic testing in the clinical practice and research of early-onset Parkinsonian disorders in a Hungarian cohort: increasing challenge in genetic counselling, improving chances in stratification for clinical trials. *Front. Genet.* 10, 1061. doi: 10.3389/fgene.2019.01061.

Ishikawa, A., Takahashi, H., Tanaka, H., Hayashi, T., and Tsuji, S. (1997). Clinical features of familial diffuse Lewy body disease. *Eur. Neurol.* 38 Suppl 1, 34-38. doi: 10.1159/000113459.

Itokawa, K., Sekine, T., Funayama, M., Tomiyama, H., Fukui, M., Yamamoto, T., et al. (2013). A case of α-synuclein gene duplication presenting with head-shaking movements. *Mov. Disord.* 28, 384-387. doi: 10.1002/mds.25243.

Kara, E., Kiely, A. P., Proukakis, C., Giffin, N., Love, S., Hehir, J., et al. (2014). A 6.4 Mb duplication of the α-synuclein locus causing frontotemporal dementia and Parkinsonism: phenotype-genotype correlations. *JAMA Neurol.* 71, 1162-1171. doi: 10.1001/jamaneurol.2014.994.

Kessler, C., Atasu, B., Hanagasi, H., Simón-Sánchez, J., Hauser, A. K., Pak, M., et al. (2018). Role of LRRK2 and SNCA in autosomal dominant Parkinson’s disease in Turkey. *Parkinsonism Relat. Disord.* 48, 34-39. doi: 10.1016/j.parkreldis.2017.12.007.

Keyser, R. J., Lombard, D., Veikondis, R., Carr, J., and Bardien, S. (2010). Analysis of exon dosage using MLPA in South African Parkinson’s disease patients. *Neurogenetics* 11, 305-312. doi: 10.1007/s10048-009-0229-6.

Ki, C. S., Stavrou, E. F., Davanos, N., Lee, W. Y., Chung, E. J., Kim, J. Y., et al. (2007). The Ala53Thr mutation in the alpha-synuclein gene in a Korean family with Parkinson disease. *Clin. Genet.* 71, 471-473. doi: 10.1111/j.1399-0004.2007.00781.x.

Kiely, A. P., Ling, H., Asi, Y. T., Kara, E., Proukakis, C., Schapira, A. H., et al. (2015). Distinct clinical and neuropathological features of G51D SNCA mutation cases compared with SNCA duplication and H50Q mutation. *Mol. Neurodegener.* 10, 41. doi: 10.1186/s13024-015-0038-3.

Kiely, A. P., Asi, Y. T., Kara, E., Limousin, P., Ling, H., Lewis, P., et al. (2013). α-Synucleinopathy associated with G51D SNCA mutation: a link between Parkinson’s disease and multiple system atrophy? *Acta Neuropathol.* 125, 753-769. doi: 10.1007/s00401-013-1096-7.

Kim, H. J., Park, G., Jeon, B. S., Park, W. Y., and Kim, Y. E. (2013). A mir-153 binding site variation in SNCA in a patient with Parkinson’s disease. *Mov. Disord.* 28, 1755-1756. doi: 10.1002/mds.25505.

Kojovic, M., Sheerin, U. M., Rubio-Agusti, I., Saha, A., Bras, J., Gibbons, V., et al. (2012). Young-onset parkinsonism due to homozygous duplication of α-synuclein in a consanguineous family. *Mov. Disord.* 27, 1827-1829. doi: 10.1002/mds.25199.

Konno, T., Ross, O. A., Puschmann, A., Dickson, D. W., and Wszolek, Z. K. (2016). Autosomal dominant Parkinson’s disease caused by SNCA duplications. *Parkinsonism Relat. Disord.* 22 Suppl 1, S1-S6. doi: 10.1016/j.parkreldis.2015.09.007.

Krüger, R., Kuhn, W., Leenders, K. L., Sprengelmeyer, R., Müller, T., Woitalla, D., et al. (2001). Familial parkinsonism with synuclein pathology: clinical and PET studies of A30P mutation carriers. *Neurology* 56, 1355-1362. doi: 10.1212/wnl.56.10.1355.

Krüger, R., Kuhn, W., Müller, T., Woitalla, D., Graeber, M., Kösel, S., et al. (1998). Ala30Pro mutation in the gene encoding alpha-synuclein in Parkinson’s disease. *Nat. Genet.* 18, 106-108. doi: 10.1038/ng0298-106.

Lesage, S., Anheim, M., Letournel, F., Bousset, L., Honoré, A., Rozas, N., et al. (2013). G51D α-synuclein mutation causes a novel parkinsonian-pyramidal syndrome. *Ann. Neurol.* 73, 459-471. doi: 10.1002/ana.23894.

Liu, H., Koros, C., Strohäker, T., Schulte, C., Bozi, M., Varvaresos, S., et al. (2021). A novel SNCA A30G mutation causes familial Parkinson’s disease. *Mov. Disord*. doi: 10.1002/mds.28534.

Markopoulou, K., Dickson, D. W., McComb, R. D., Wszolek, Z. K., Katechalidou, L., Avery, L., et al. (2008). Clinical, neuropathological and genotypic variability in SNCA A53T familial Parkinson’s disease. Variability in familial Parkinson’s disease. *Acta Neuropathol.* 116, 25-35. doi: 10.1007/s00401-008-0372-4.

Markopoulou, K., Wszolek, Z. K., Pfeiffer, R. F., and Chase, B. A. (1999). Reduced expression of the G209A alpha-synuclein allele in familial Parkinsonism. *Ann. Neurol.* 46, 374-381. doi: 10.1002/1531-8249(199909)46:3<374::aid-ana13>3.0.co;2-9.

Markopoulou, K., Wszolek, Z. K., and Pfeiffer, R. F. (1995). A Greek-American kindred with autosomal dominant, levodopa-responsive parkinsonism and anticipation. *Ann. Neurol.* 38, 373-378. doi: 10.1002/ana.410380306.

Martikainen, M. H., Päivärinta, M., Hietala, M., and Kaasinen, V. (2015). Clinical and imaging findings in Parkinson disease associated with the A53E SNCA mutation. *Neurol. Genet.* 1, e27. doi: 10.1212/NXG.0000000000000027.

Meeus, B., Verstraeten, A., Crosiers, D., Engelborghs, S., Van den Broeck, M., Mattheijssens, M., et al. (2012). DLB and PDD: a role for mutations in dementia and Parkinson disease genes? *Neurobiol. Aging* 33, 629.e5-629.e18. doi: 10.1016/j.neurobiolaging.2011.10.014.

Michell, A. W., Barker, R. A., Raha, S. K., and Raha-Chowdhury, R. (2005). A case of late onset sporadic Parkinson’s disease with an A53T mutation in alpha-synuclein. *J. Neurol. Neurosurg. Psychiatry* 76, 596-597. doi: 10.1136/jnnp.2004.046425.

Morfis, L., and Cordato, D. J. (2006). Dementia with Lewy bodies in an elderly Greek male due to alpha-synuclein gene mutation. *J. Clin. Neurosci.* 13, 942-944. doi: 10.1016/j.jocn.2005.11.040.

Muenter, M. D., Forno, L. S., Hornykiewicz, O., Kish, S. J., Maraganore, D. M., Caselli, R. J., et al. (1998). Hereditary form of parkinsonism-dementia. *Ann. Neurol.* 43, 768-781. doi: 10.1002/ana.410430612.

Mutez, E., Leprêtre, F., Le Rhun, E., Larvor, L., Duflot, A., Mouroux, V., et al. (2011). SNCA locus duplication carriers: from genetics to Parkinson disease phenotypes. *Hum. Mutat.* 32, E2079-E2090. doi: 10.1002/humu.21459.

Nan, H., Takaki, R., Maruyama, T., Baba, Y., Ohara, S., Shindo, K., et al. (2020). Orthostatic hypotension as a core symptom in a Japanese family harboring SNCA duplication. *Parkinsonism Relat. Disord.* 81, 28-30. doi: 10.1016/j.parkreldis.2020.10.006.

Nishioka, K., Hayashi, S., Farrer, M. J., Singleton, A. B., Yoshino, H., Imai, H., et al. (2006). Clinical heterogeneity of alpha-synuclein gene duplication in Parkinson’s disease. *Ann. Neurol.* 59, 298-309. doi: 10.1002/ana.20753.

Nishioka, K., Ross, O. A., Ishii, K., Kachergus, J. M., Ishiwata, K., Kitagawa, M., et al. (2009). Expanding the clinical phenotype of SNCA

duplication carriers. *Mov. Disord.* 24, 1811-1819. doi: 10.1002/mds.22682.
Nuytemans, K., Meeus, B., Crosiers, D., Brouwers, N., Goossens, D., Engelborghs, S., et al. (2009). Relative contribution of simple mutations vs. copy number variations in five Parkinson disease genes in the Belgian population. *Hum. Mutat.* 30, 1054-1061. doi: 10.1002/humu.21007.
Olgiati, S., Thomas, A., Quadri, M., Breedveld, G. J., Graafland, J., Eussen, H., et al. (2015). Early-onset parkinsonism caused by alpha-synuclein gene triplication: clinical and genetic findings in a novel family. *Parkinsonism Relat. Disord.* 21, 981-986. doi: 10.1016/j.parkreldis.2015.06.005.
Papadimitriou, A., Veletza, V., Hadjigeorgiou, G. M., Patrikiou, A., Hirano, M., and Anastasopoulos, I. (1999). Mutated alpha-synuclein gene in two Greek kindreds with familial PD: incomplete penetrance? *Neurology* 52, 651-654. doi: 10.1212/wnl.52.3.651.
Papapetropoulos, S., Paschalis, C., Athanassiadou, A., Papadimitriou, A., Ellul, J., Polymeropoulos, M. H., et al. (2001). Clinical phenotype in patients with alpha-synuclein Parkinson’s disease living in Greece in comparison with patients with sporadic Parkinson’s disease. *J. Neurol. Neurosurg. Psychiatry* 70, 662-665. doi: 10.1136/jnnp.70.5.662.
Pasanen, P., Palin, E., Pohjolan-Pirhonen, R., Pöyhönen, M., Rinne, J. O., Päivärinta, M., et al. (2017). SNCA mutation p.Ala53Glu is derived from a common founder in the Finnish population. *Neurobiol. Aging* 50, 168.e5-168.e8. doi: 10.1016/j.neurobiolaging.2016.10.014.
Pasanen, P., Myllykangas, L., Siitonen, M., Raunio, A., Kaakkola, S., Lyytinen, J., et al. (2014). Novel α-synuclein mutation A53E associated with atypical multiple system atrophy and Parkinson’s disease-type pathology. *Neurobiol. Aging* 35, 2180.e1-2180.e5. doi: 10.1016/j.neurobiolaging.2014.03.024.
Pimentel, M. M., Rodrigues, F. C., Leite, M. A., Campos Júnior, M., Rosso, A. L., Nicaretta, D. H., et al. (2015). Parkinson disease: α-synuclein mutational screening and new clinical insight into the p.E46K mutation. *Parkinsonism Relat. Disord.* 21, 586-589. doi: 10.1016/j.parkreldis.2015.03.011.
Polymeropoulos, M. H., Lavedan, C., Leroy, E., Ide, S. E., Dehejia, A., Dutra, A., et al. (1997). Mutation in the alpha-synuclein gene identified in families with Parkinson’s disease. *Science* 276, 2045-2047. doi: 10.1126/science.276.5321.2045.
Proukakis, C., Dudzik, C. G., Brier, T., MacKay, D. S., Cooper, J. M., Millhauser, G. L., et al. (2013). A novel α-synuclein missense mutation in Parkinson disease. *Neurology* 80, 1062-1064. doi: 10.1212/WNL.0b013e31828727ba.
Puschmann, A., Jiménez-Ferrer, I., Lundblad-Andersson, E., Mårtensson, E., Hansson, O., Odin, P., et al. (2019). Low prevalence of known pathogenic mutations in dominant PD genes: a Swedish multicenter study. *Parkinsonism Relat. Disord.* 66, 158-165. doi: 10.1016/j.parkreldis.2019.07.032.
Puschmann, A., Ross, O. A., Vilariño-Güell, C., Lincoln, S. J., Kachergus, J. M., Cobb, S. A., et al. (2009). A Swedish family with de novo alpha-synuclein A53T mutation: evidence for early cortical dysfunction. *Parkinsonism Relat. Disord.* 15, 627-632. doi: 10.1016/j.parkreldis.2009.06.007.
Ricciardi, L., Petrucci, S., Di Giuda, D., Serra, L., Spanò, B., Sensi, M., et al. (2016). The Contursi family 20 years later: intrafamilial

phenotypic variability of the SNCA p.A53T mutation. *Mov. Disord.* 31, 257-258. doi: 10.1002/mds.26549.
Robak, L. A., Du, R., Yuan, B., Gu, S., Alfradique-Dunham, I., Kondapalli, V., et al. (2020). Integrated sequencing and array comparative genomic hybridization in familial Parkinson disease. *Neurol. Genet.* 6, e498. doi: 10.1212/NXG.0000000000000498.
Ross, O. A., Braithwaite, A. T., Skipper, L. M., Kachergus, J., Hulihan, M. M., Middleton, F. A., et al. (2008). Genomic investigation of alpha-synuclein multiplication and parkinsonism. *Ann. Neurol.* 63, 743-750. doi: 10.1002/ana.21380.
Scott, W. K., Yamaoka, L. H., Stajich, J. M., Scott, B. L., Vance, J. M., Roses, A. D., et al. (1999). The alpha-synuclein gene is not a major risk factor in familial Parkinson disease. *Neurogenetics* 2, 191-192. doi: 10.1007/s100480050083.
Sekine, T., Kagaya, H., Funayama, M., Li, Y., Yoshino, H., Tomiyama, H., et al. (2010). Clinical course of the first Asian family with Parkinsonism related to SNCA triplication. *Mov. Disord.* 25, 2871-2875. doi: 10.1002/mds.23313.
Seo, S. H., Bacolla, A., Yoo, D., Koo, Y. J., Cho, S. I., Kim, M. J., et al. (2020). Replication-based rearrangements are a common mechanism for SNCA duplication in Parkinson's disease. *Mov. Disord.* 35, 868-876. doi: 10.1002/mds.27998.
Shin, C. W., Kim, H. J., Park, S. S., Kim, S. Y., Kim, J. Y., and Jeon, B. S. (2010). Two Parkinson's disease patients with alpha-synuclein gene duplication and rapid cognitive decline. *Mov. Disord.* 25, 957-959. doi: 10.1002/mds.23043.
Singleton, A., Gwinn-Hardy, K., Sharabi, Y., Li, S. T., Holmes, C., Dendi, R., et al. (2004). Association between cardiac denervation and parkinsonism caused by alpha-synuclein gene triplication. *Brain* 127, 768-772. doi: 10.1093/brain/awh081.
Singleton, A. B., Farrer, M., Johnson, J., Singleton, A., Hague, S., Kachergus, J., et al. (2003). alpha-Synuclein locus triplication causes Parkinson's disease. *Science* 302, 841. doi: 10.1126/science.1090278.
Sironi, F., Trotta, L., Antonini, A., Zini, M., Ciccone, R., Della Mina, E., et al. (2010). alpha-Synuclein multiplication analysis in Italian familial Parkinson disease. *Parkinsonism Relat. Disord.* 16, 228-231. doi: 10.1016/j.parkreldis.2009.09.008.
Somme, J. H., Gomez-Esteban, J. C., Molano, A., Tijero, B., Lezcano, E., and Zarranz, J. J. (2011). Initial neuropsychological impairments in patients with the E46K mutation of the α-synuclein gene (PARK 1). *J. Neurol. Sci.* 310, 86-89. doi: 10.1016/j.jns.2011.07.047.
Spira, P. J., Sharpe, D. M., Halliday, G., Cavanagh, J., and Nicholson, G. A. (2001). Clinical and pathological features of a Parkinsonian syndrome in a family with an Ala53Thr alpha-synuclein mutation. *Ann. Neurol.* 49, 313-319. doi: 10.1002/ana.67.
Takamura, S., Ikeda, A., Nishioka, K., Furuya, H., Tashiro, M., Matsushima, T., et al. (2016). Schizophrenia as a prodromal symptom in a patient harboring SNCA duplication. *Parkinsonism Relat. Disord.* 25, 108-109. doi: 10.1016/j.parkreldis.2016.01.028.
Tambasco, N., Nigro, P., Romoli, M., Prontera, P., Simoni, S., and Calabresi, P. (2016). A53T in a parkinsonian family: a clinical update of the SNCA phenotypes. *J. Neural. Transm. (Vienna)* 123, 1301-1307. doi: 10.1007/s00702-016-1578-6.
Tijero, B., Gómez-Esteban, J. C., Lezcano, E., Fernández-González, C., Somme, J., Llorens, V., et al. (2013). Cardiac sympathetic denervation in symptomatic and asymptomatic carriers of the E46K mutation in the α synuclein gene. *Parkinsonism Relat. Disord.* 19, 95-100. doi: 10.1016/j.parkreldis.2012.08.001.

Tokutake, T., Ishikawa, A., Yoshimura, N., Miyashita, A., Kuwano, R., Nishizawa, M., et al. (2014). Clinical and neuroimaging features of patient with early-onset Parkinson's disease with dementia carrying SNCA p.G51D mutation. *Parkinsonism Relat. Disord.* 20, 262-264. doi: 10.1016/j.parkreldis.2013.11.008.

Troiano, A. R., Cazeneuve, C., Le Ber, I., Bonnet, A. M., Lesage, S., and Brice, A. (2008). Re: Alpha-synuclein gene duplication is present in sporadic Parkinson disease. *Neurology* 71, 1295. doi: 10.1212/01.wnl.0000338435.78120.0f.

Uchiyama, T., Ikeuchi, T., Ouchi, Y., Sakamoto, M., Kasuga, K., Shiga, A., et al. (2008). Prominent psychiatric symptoms and glucose hypometabolism in a family with a SNCA duplication. *Neurology* 71, 1289-1291. doi: 10.1212/01.wnl.0000327607.28928.e6.

Wang, L., Nuytemans, K., Bademci, G., Jauregui, C., Martin, E. R., Scott, W. K., et al. (2013). High-resolution survey in familial Parkinson disease genes reveals multiple independent copy number variation events in PARK2. *Hum. Mutat.* 34, 1071-1074. doi: 10.1002/humu.22344.

Xiong, W. X., Sun, Y. M., Guan, R. Y., Luo, S. S., Chen, C., An, Y., et al. (2016). The heterozygous A53T mutation in the alpha-synuclein gene in a Chinese Han patient with Parkinson disease: case report and literature review. *J. Neurol.* 263, 1984-1992. doi: 10.1007/s00415-016-8213-1.

Yoshino, H., Hirano, M., Stoessl, A. J., Imamichi, Y., Ikeda, A., Li, Y., et al. (2017). Homozygous alpha-synuclein p.A53V in familial Parkinson's disease. *Neurobiol. Aging* 57, 248.e7-248.e12. doi: 10.1016/j.neurobiolaging.2017.05.022.

Youn, J., Lee, C., Oh, E., Park, J., Kim, J. S., Kim, H. T., et al. (2019). Genetic variants of PARK genes in Korean patients with early-onset Parkinson's disease. *Neurobiol. Aging* 75, 224.e9-224.e15. doi: 10.1016/j.neurobiolaging.2018.10.030.

Zafar, F., Valappil, R. A., Kim, S., Johansen, K. K., Chang, A. L. S., Tetrud, J. W., et al. (2018). Genetic fine-mapping of the Iowan SNCA gene triplication in a patient with Parkinson's disease. *NPJ Parkinsons Dis.* 4, 18. doi: 10.1038/s41531-018-0054-4.

Zarranz, J. J., Fernández-Bedoya, A., Lambarri, I., Gómez-Esteban, J. C., Lezcano, E., Zamacona, J., et al. (2005). Abnormal sleep architecture is an early feature in the E46K familial synucleinopathy. *Mov. Disord.* 20, 1310-1315. doi: 10.1002/mds.20581.

Zarranz, J. J., Alegre, J., Gómez-Esteban, J. C., Lezcano, E., Ros, R., Ampuero, I., et al. (2004). The new mutation, E46K, of alpha-synuclein causes Parkinson and Lewy body dementia. *Ann. Neurol.* 55, 164-173. doi: 10.1002/ana.10795.

Zhao, Y., Qin, L., Pan, H., Liu, Z., Jiang, L., He, Y., et al. (2020). The role of genetics in Parkinson's disease: a large cohort study in Chinese mainland population. *Brain* 143, 2220-2234. doi: 10.1093/brain/awaa167.

Supplementary Table 3. Associations between different types of *SNCA* gene variants and recorded psychiatric signs as well as cognitive decline/dementia.

**Clinical features**	**Point variant (n = 134)**	**Multiplication (n = 97)**	**χ^2 value**	***P*-value**
Psychiatric signs	47 (35.07%)	52 (53.61%)	7.892	**0.005**
Cognitive decline/dementia	48 (35.82%)	54 (55.67%)	8.991	**0.003**

SNCA, the alpha-synuclein gene.
